# Persistence of SARS-CoV-2 in saliva: Implications for late-stage diagnosis and infectious duration

**DOI:** 10.1371/journal.pone.0282708

**Published:** 2023-03-16

**Authors:** Abby Chopoorian, Padmapriya Banada, Robert Reiss, David Elson, Samuel Desind, Claire Park, Sukalyani Banik, Emily Hennig, Aanchal Wats, Austin Togba, Abraham Wei, Naranjargal Daivaa, Laura Palo, Mitchell Hirsch, Carter Campbell, Pooja Saiganesh, David Alland, Yingda L. Xie

**Affiliations:** 1 Department of Medicine, Public Health Research Institute, Rutgers New Jersey Medical School, Newark, New Jersey, United States of America; 2 School of Medicine, Rutgers New Jersey Medical School, Newark, New Jersey, United States of America; 3 Rutgers School of Public Health, Piscataway, New Jersey, United States of America; AIIMS: All India Institute of Medical Sciences, INDIA

## Abstract

Saliva has been a COVID-19 diagnostic specimen of interest due to its simple collection, scalability, and yield. Yet COVID-19 testing and estimates of the infectious period remain largely based on nasopharyngeal and nasal swabs. We sought to evaluate whether saliva testing captured prolonged presence of SARS-CoV-2 and potential infectiousness later in the disease course. We conducted an observational study of symptomatic COVID-19 patients at University Hospital in Newark, NJ. Paired saliva and nasal specimens from 96 patients were analyzed, including longitudinal analysis of paired observations from 28 of these patients who had multiple time-points. Saliva detected significantly more cases of COVID-19 beyond 5 days (86.1% [99/115] saliva vs 48.7% [56/115] nasal, p-value < 0.001), 9 days (79.4% [50/63] saliva vs 36.5% [23/63] nasal, p-value < 0.001) and 14 days (71.4% [20/28] saliva vs 32.1% [9/28] nasal, p-value = 0.010) of symptoms. Additionally, saliva yielded lower cycle thresholds across all time periods, indicative of higher viral loads in saliva. In the longitudinal analysis, a log-rank analysis indicated that the survival curve for saliva was significantly different from the curve for nasal swabs (p<0.001) with a median survival time for saliva of 18 days compared to 13 days for nasal swabs. We additionally performed saliva viral cultures among a similar COVID-19 patient cohort and noted patients with positive saliva viral cultures between 7 to 28 days of symptoms. Findings from this study suggest that SARS-CoV-2 RNA persists longer and in higher abundance in saliva compared to nasal swabs, with potential of prolonged propagating virus. Testing saliva may thus increase yield for detecting potentially infectious virus even beyond the first five days of symptomatic COVID-19.

## Introduction

Rapid and accurate diagnosis has been critical to the management of the COVID-19 pandemic. Saliva and nasal swabs are non-invasive methods for SARS-CoV-2 testing with minimal or no supervision that have enabled scale-up of COVID-19 testing. A decline in viral shedding [1–4] as well as loss of culturable virus in the first week of symptoms [5–8], have primarily been observed in studies of pharyngeal and nasal specimens. However, the dynamics of early viral shedding from saliva can differ from nasal specimens [9], guiding preferences for testing strategies at different stages of infection. These differences are also important in late infection, including frequent scenarios where patients seek medical attention or test later during their disease course. However, few studies have compared the length of time that SARS-CoV-2 is detectable in saliva compared to nasal swabs beyond the first week of symptoms when false-negative tests and missed diagnoses are higher [1].

SARS-CoV-2 can independently colonize the oral cavity and/or salivary glands via several pathways [10, 11] with transmission through saliva droplets perpetuated by activities such as speaking and sneezing [12]. PCR testing of saliva has been shown to have comparable [13–15] or higher sensitivity [16] and stability [13] than PCR testing for COVID-19 using nasal or nasopharyngeal (NP) swabs. We posited that SARS-CoV-2 virus might be detectable in saliva for longer periods than nasal swabs, including during the late post-symptomatic period. Such prolonged positivity in saliva would suggest that saliva-based tests might identify more individuals with COVID-19, especially for those who were not tested within the first 5 days of symptoms. These findings could also change our understanding of the SARS-CoV-2 infectious period and the potential need for follow-up saliva testing. To determine whether saliva-based diagnostics would add value for SARS-CoV-2 detection and infection control, we evaluated longitudinal data from COVID-19 patients in New Jersey to compare the longevity of detectable virus from saliva versus nasal swabs.

## Methods

### Study population

To compare viral load dynamics between saliva and nasal swabs, we conducted a substudy within a prospective observational cohort study of diagnostic and therapeutic strategies among COVID-19 patients. This study was approved by the Rutgers University IRB for human subject research (Rutgers IRB #Pro2020001138). Participants were enrolled from patients at University Hospital in Newark, New Jersey. Eligible patients in the parent study were 18 years of age or older who tested positive for COVID-19 by an NP swab using the hospital PCR test, most commonly Simplexa COVID-19 Direct EUA (Diasorin Molecular LLC, Cypress, CA), were able to consent, not prisoners, and willing and able to provide respiratory specimens including saliva and nasal swabs. Participants who were planned for COVID-19 treatment during their admission were followed throughout their hospitalization. All parent study participants with a known time from symptom onset and who provided both a saliva and nasal specimen for at least one study timepoint were included in the sub-study. Individual time points were excluded if the saliva and/or nasal swab specimen was missing, precluding a paired analysis at that timepoint.

All eligible participants were included in the overall analysis, in which paired saliva-nasal swab observations were referenced to time of symptom onset. For a time-to-event analysis of conversion to SARS-CoV-2 negative for each specimen type, we further excluded participants in the overall analysis cohort with only a single time-point to derive a “longitudinal analysis cohort” consisting only of individuals with multiple time-points.

### Specimen collection

Specimens were collected by trained study personnel. All patients were instructed to self-collect saliva by clearing their throat and spitting carefully into a sterile, wide-mouthed container. All specimens more than 0.5 mL were accepted and included in the study. Anterior nasal swabs were collected by rotating a flocked nylon tip swab (Copan Diagnostics, Murietta, CA) 1 cm inside each nostril for 10–15 seconds. Nasopharyngeal (NP) swabs were collected according to the CDC guideline [17] with a thinner nylon tip swab (Copan Diagnostics, Murietta, CA) at only the first (baseline) timepoint. The nasal and NP swabs were placed immediately into 3mL of viral transport medium (VTM; Labscoop, Little Rock, AR). An additional nasal swab specimen was placed in 3mL of eNAT medium (Copan Diagnostics, Murrieta, CA) and, at the laboratory, swabs of direct saliva were also placed in 3mL of VTM or eNAT medium (Copan Diagnostics, Murrieta, CA). All participants were asked to provide at least one set of specimens. Among participants who continued to stay in the hospital for COVID-19 management, specimen collection was attempted every 2–3 days for the duration of their admission until they were discharged or ended their participation, whichever occurred first.

### Specimen transport and testing

Specimens were transported to the Public Health Research Institute in Newark, NJ from the University Hospital in double boxed specimen transfer containers and tested using the Cepheid Xpert Xpress SARS-CoV-2 assay (‘Xpert’; Cepheid, Sunnyvale, CA) following the company recommended protocol. In brief, all specimens were tested by transferring 300μl of the specimen (direct saliva, nasal swab in VTM, NP swab in VTM, nasal swab in eNAT, or saliva swab in eNAT) directly into the Xpert cartridge. Xpert cycle threshold (Ct) values were analyzed based on the N gene target, which codes for the viral nucleocapsid. Negative tests were represented as Ct = 50.

### Saliva culture study

To explore whether detection of viral RNA in saliva beyond the first week of symptoms could ever signify infectious virus, we analyzed results from a separate study to investigate the relationship between the Ct value of the N gene target in the Xpert Xpress test and viral culture positivity (Rutgers IRB # Pro2020003236). Virus cultures were performed using the Median Tissue Culture Infectious Dose assay (TCID50) method [18, 19] using specimens from a separate but similar UH adult patient cohort with COVID-19 within 72 hours of a positive NP swab RT-PCR test.

### Statistical analysis

Median Ct values are presented with bias-corrected and accelerated 95% confidence intervals generated via bootstrapping. Comparative analysis of median Ct values is achieved through ANOVA/ Wilcoxon signed-rank test for clustered data [19] and the chi-square test. A Ct value of less than 30 has been associated with SARS-CoV-2 viral culture positivity [5, 20], and thus proportions of specimens with Ct values less than 30 were compared between specimen types by time period using the Obuchowski variation of the McNemar test for correlated proportions [21].

The Kaplan-Meier method was used to derive the survival function and plot the survival curves for each specimen type from the longitudinal analysis cohort. Survival curves were compared using the log-rank test. A cox proportional hazards regression model was fitted to the data with estimation of the standard error to account for paired observations, and the regression coefficient for specimen type was estimated. Estimates were considered statistically significant if the associated p-value was less than or equal to 0.05. All analyses were conducted with R Statistical Software version 4.0.5 (R Foundation for Statistical Computing, Vienna, Austria).

## Results

### Participant selection

**Fig 1:**
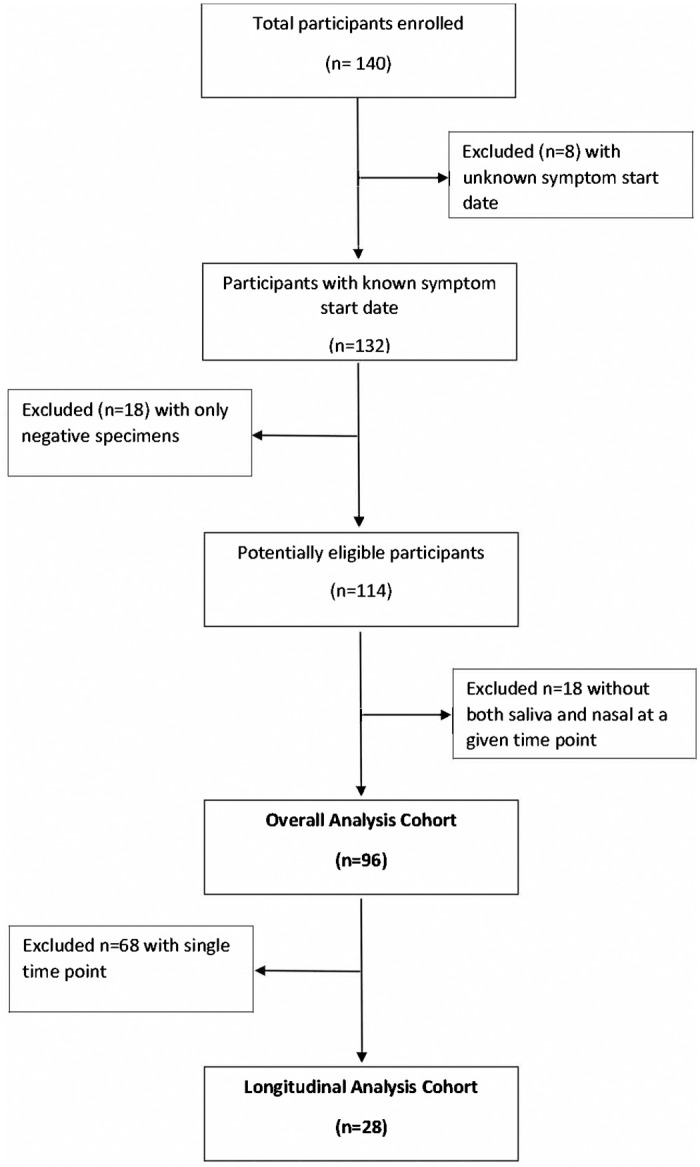
Study flowchart.

Between June 2020 and August 2021, 140 participants were enrolled into the study, of whom n = 44 were excluded (8 with unknown symptom start dates, 18 with only negative test results at all time points, and 18 without any paired observation of saliva and nasal specimens) (Fig 1). From the remaining 96 participants, 147 saliva and nasal swabs were analyzed as paired observations in the overall analysis. From these 96 participants in the overall analysis cohort, a total of 28 participants with more than one time point collection were included for the longitudinal analysis.

### Overall analysis

#### Baseline participant characteristics

Baseline characteristics of the overall and longitudinal cohorts are shown in Table 1. Among the N = 96 in the overall analysis cohort, average patient age was 54.7 years, 53% were male, and 55% were Hispanic. On average, the baseline specimen collection occurred 7 days after symptom onset. Most participants had at least one comorbidity, commonly hypertension and obesity (53% and 50%, respectively), reported exposure to another person with COVID-19 symptoms (59%), or received supplemental oxygen during their hospitalization (66%).

**Table 1:** Characteristics of participants in the overall and longitudinal cohorts.

	Overall analysis cohort	Longitudinal cohort
	(N = 96)	(n = 28)
**Mean Age in years (SD)**	54.7 (14.6)	56.8 (14.9)
Symptom duration prior to baseline collection		
**Mean (range)**	7.0 days (-1–21)	8.6 days (2–21)
Days between in-hospital NP swab PCR and baseline collection: **mean (range)**	1.8 days (-4–7)	1.7 days (0–6)
Number of follow-up time-points per participant: **mean (range)**	0.5 timepoints	1.8 timepoints
	(0–7)	(1–7)
**# Male (%)**	51 (53%)	18 (64%)
**# Female (%)**	45 (47%)	10 (36%)
**Ethnicity (%)**		
Hispanic	53 (55%)	17 (61%)
Black	36 (38%)	7 (25%)
White	4 (4%)	2 (7%)
More than one race/Other	3 (3%)	2 (7%)
**Comorbidities**		
Hypertension	51 (53%)	14 (50%)
Obesity	48 (50%)	12 (43%)
Diabetes Mellitus	33 (34%)	10 (36%)
Lung Disease (e.g., COPD)	12 (13%)	5 (18%)
Chronic Heart Disease	10 (10%)	4 (14%)
Chronic Kidney Disease	7 (7%)	0 (0%)
HIV	2 (2%)	0 (0%)
Other	41 (43%)	9 (32%)
No chronic disease	18 (19%)	3 (11%)
**Smoking (%)**		
Yes	10 (10%)	1 (4%)
No	86 (90%)	27 (96%)
**COVID Vaccination History (%)**		
Yes (1 dose)	3 (3%)	0 (0%)
Yes (2 doses)	5 (5%)	2 (7%)
No	68 (71%)	24 (86%)
Unknown/Missing	20 (21%)	2 (7%)
**Sick Contacts (%)**		
Yes	57 (59%)	21 (75%)
No	34 (35%)	7 (25%)
Missing	5 (6%)	0 (0%)
**COVID symptoms (%)**		
Cough	60 (63%)	18 (64%)
Shortness of breath/difficulty breathing	66 (69%)	21 (75%)
Fever	53 (55%)	16 (57%)
Diarrhea	24 (25%)	8 (29%)
Chest Pain	24 (25%)	8 (29%)
Chills	32 (33%)	8 (29%)
Sore throat	6 (6%)	2 (7%)
Nausea/vomiting	28 (29%)	6 (21%)
**Oxygen Support Required (%)** ^a^		
None	33 (34%)	2 (7%)
Nasal Cannula	62 (65%)	26 (93%)
Non-rebreather	5 (5%)	3 (11%)
Non-Invasive Mechanical Ventilation	8 (8%)	2 (7%)
Intubation	3 (3%)	1 (4%)
Other	1 (1%)	0 (0%)
**COVID-directed Treatment (%)** ^a^		
Antiviral ^b^	71 (74%)	25 (89%)
Anti-inflammatory ^c^	60 (63%)	24 (86%)
Immunotherapy ^d^	6 (6%)	2 (7%)
No Treatment	18 (19%)	2 (7%)

^a^ At any point during hospitalization.

^**b**^ Antiviral treatments include hydroxychloroquine and remdesivir.

^**c**^ Anti-inflammatory treatments include ruxolitinib, tocilizumab and zanabrutinib.

^**d**^ Immunotherapies include convalescent plasma and monoclonal antibodies.

Participant characteristics were also compared across time periods (Table 2). Time intervals of interest were beyond that of 5 days, representing the minimum recommended isolation period for COVID-19 at the time of writing [22], and beyond 9 days when individuals are generally considered no longer infectious due to lack of culturable virus from nasal or nasopharyngeal samples [4, 23]. Dyspnea appeared to be more common in the 5–9 and 10–14 day interval groups, but all other symptoms were similar between groups. Participants in later time periods were more likely to require oxygen support than those in earlier periods. Additionally, the patients who were enrolled less than 5 days from symptom onset were more likely to report their race or ethnicity as Black, report smoking, and had a higher prevalence of chronic kidney disease (Table 2).

**Table 2:** Characteristics of participants in the overall analysis cohort by days from symptom onset.

	Less than 5 days (n = 21)	5 to 9 days (n = 54)	10 to 14 days (n = 29)	15 or more days (n = 15)
**Mean Age in years (SD)**	55.4 (13.6)	53.7 (15.5)	56.5 (15.1)	56.3 (13.6)
Symptom duration: **Mean in days (range)**	2.5 (-1–4)	6.5 (2–9)	7.9 (2–13)	13.3 (4–21)
Days between in-hospital NP swab PCR and baseline collection: **mean (range)**	1.1 (-4–5)	2.1 (0–6)	1.9 (0–7)	1.5 (-3–6)
Number of follow-up time-points per participant: **mean (range)**	0.6 (0–7)	0.5 (0–7)	1.5 (0–7)	1.9 (0–7)
**# Male (%)**	13 (62%)	31 (57%)	15 (52%)	8 (53%)
**# Female (%)**	8 (38%)	23 (43%)	14 (48%)	7 (47%)
**Ethnicity (%)**				
Hispanic	6 (29%)	35 (65%)	16 (55%)	9 (60%)
Black	15 (71%)	15 (28%)	12 (41%)	3 (20%)
White	0 (0%)	4 (7%)	1 (3%)	0 (0%)
More than one race/Other	0 (0%)	0 (0%)	0 (0%)	3 (20%)
**Comorbidities**				
Hypertension	12 (57%)	27 (50%)	12 (41%)	9 (60%)
Obesity	8 (38%)	24 (44%)	14 (48%)	10 (67%)
Diabetes Mellitus	9 (43%)	17 (31%)	8 (28%)	5 (33%)
Lung Disease (e.g., COPD)	3 (14%)	8 (15%)	6 (21%)	1 (7%)
Chronic Heart Disease	5 (24%)	5 (9%)	2 (7%)	1 (7%)
Chronic Kidney Disease	4 (19%)	2 (4%)	1 (3%)	0 (0%)
HIV	0 (0%)	2 (4%)	0 (0%)	0 (0%)
Other	9 (43%)	22 (41%)	12 (41%)	6 (40%)
No chronic disease	4 (19%)	9 (17%)	5 (17%)	2 (13%)
**Smoking (%)**				
Yes	7 (33%)	4 (7%)	2 (7%)	1 (7%)
No	14 (67%)	50 (93%)	26 (90%)	14 (93%)
Missing	0 (0%)	0 (0%)	1 (3%)	0 (0%)
**COVID Vaccination History (%)**				
Yes (1 dose)	2 (10%)	0 (0%)	0 (0%)	0 (0%)
Yes (2 doses)	0 (0%)	1 (2%)	0 (0%)	1 (7%)
No	11 (48%)	43 (70%)	24 (79%)	11 (67%)
Missing	8 (43%)	10 (28%)	5 (21%)	3 (27%)
**Sick Contacts (%)**				
Yes	10 (48%)	34 (63%)	21 (72%)	9 (60%)
No	6 (29%)	14 (26%)	6 (21%)	4 (27%)
Missing	5 (24%)	6 (11%)	2 (7%)	2 (13%)
**COVID symptoms (%)**				
Cough	12 (57%)	37 (69%)	17 (59%)	9 (60%)
Shortness of breath	11 (52%)	42 (78%)	24 (83%)	8 (53%)
Fever	8 (38%)	34 (63%)	18 (62%)	8 (53%)
Diarrhea	4 (19%)	17 (31%)	9 (31%)	3 (20%)
Chest Pain	6 (29%)	13 (24%)	8 (28%)	4 (27%)
Chills	7 (33%)	18 (31%)	11 (38%)	5 (33%)
Sore throat	0 (0%)	3 (6%)	3 (10%)	0 (0%)
Nausea/vomiting	6 (29%)	18 (33%)	8 (28%)	2 (13%)
**Oxygen Support Required (%)** ^a^				
None	11 (53%)	19 (35%)	7 (24%)	1 (7%)
Nasal Cannula	9 (43%)	34 (63%)	21 (72%)	14 (93%)
Non-rebreather	0 (0%)	2 (4%)	0 (0%)	1 (7%)
Non-Invasive Ventilation	1 (5%)	5 (9%)	3 (10%)	0 (0%)
Intubation	0 (0%)	2 (4%)	1 (3%)	0 (0%)
Other	0 (0%)	1 (2%)	0 (0%)	0 (0%)
Missing	1 (5%)	0 (0%)	3 (7%)	0 (0%)
**COVID-directed Treatment** ^a^				
Antiviral ^b^	7 (33%)	38 (70%)	16 (55%)	9 (60%)
Anti-inflammatory ^c^	6 (29%)	34 (63%)	23 (79%)	8 (53%)
Immunotherapy ^d^	1 (5%)	1 (2%)	0 (0%)	0 (0%)
No Treatment	12 (48%)	8 (15%)	4 (14%)	5 (33%)

^a^ Status evaluated for each observation at time of specimen acquisition.

^**b**^ Antiviral treatments include hydroxychloroquine and remdesivir.

^**c**^ Anti-inflammatory treatments include ruxolitinib, tocilizumab and zanabrutinib.

^**d**^ Immunotherapies include convalescent plasma and monoclonal antibodies.

#### Nasal swabs in VTM versus direct saliva

**Fig 2:**
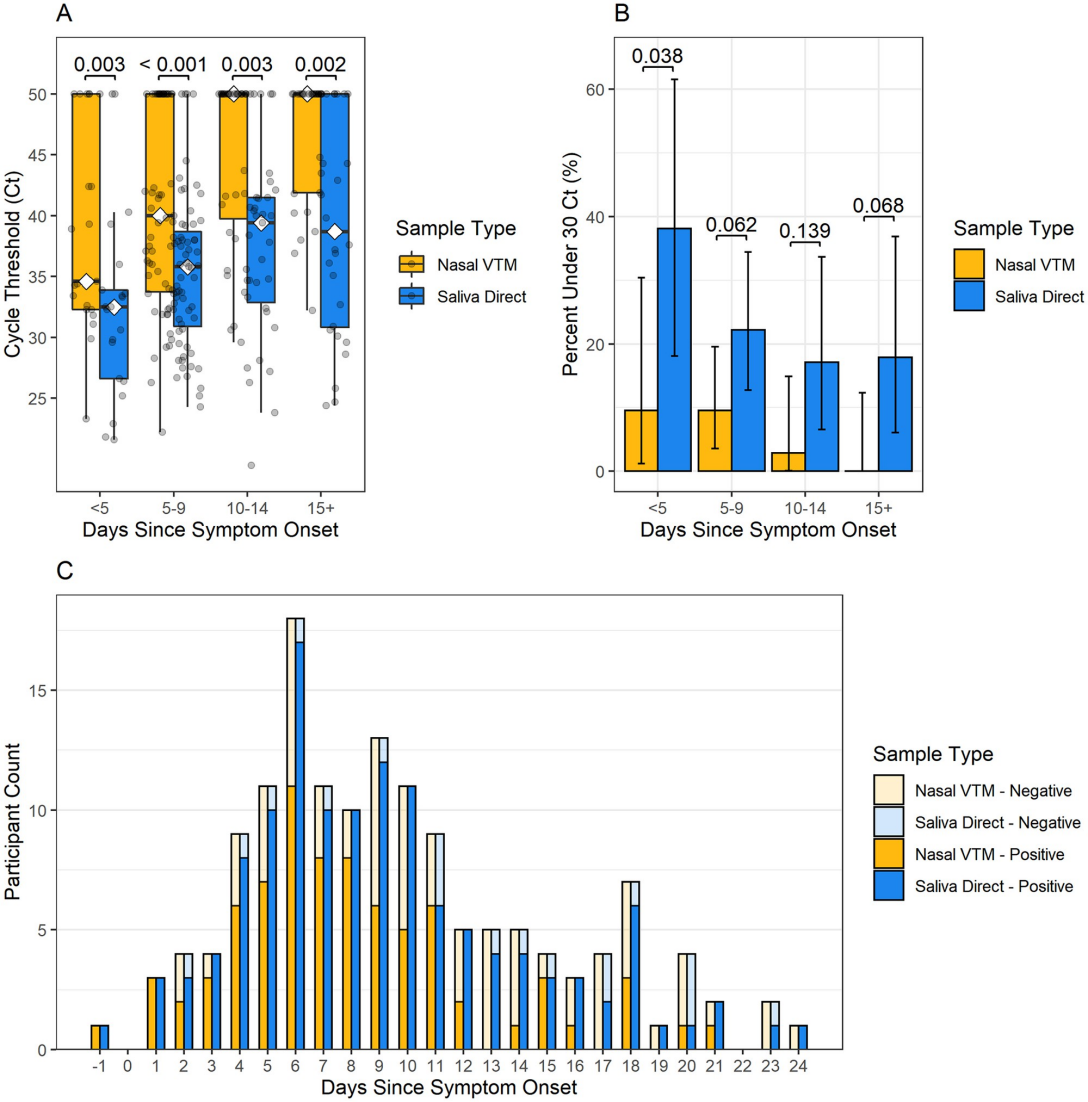
SARS-CoV-2 longevity in overall analysis cohort saliva and nasal specimens. (A) Ct values, (B) percent of Ct values under 30 and (C) number of positive and negative test results for saliva direct and nasal VTM specimens over days from symptom onset for the overall cohort (N = 96).

In the overall analysis, saliva detected significantly more cases of SARS-CoV-2 within 5 days (90.6% [29/32] saliva vs 68.8% [22/32] nasal, p = 0.009), beyond 5 days (86.1% saliva [99/115] vs 48.7% [56/115] nasal, p < 0.001), beyond 9 days (79.4% [50/63] saliva vs 36.5% [23/63] nasal, p-value < 0.001) and beyond 14 days (71.4% [20/28] saliva vs 32.1% [9/28] nasal, p-value = 0.010) of symptoms. Overall median N2-target Ct values were earlier (corresponding to higher viral load) for saliva (median Ct 36.0, 95% CI 33.8 to 37.6) than for nasal swabs (median Ct 42.4, 95% CI 40.9 to 50.0). Ct values were also earlier for saliva than for nasal swabs across all time periods, including beyond 14 days of symptoms (p = 0.002) (Fig 2A and Table 3). The percentage of N2-target Ct values that were <30 was higher in saliva (22.4%) compared to nasal swabs (6.1%) across all time points, though statistically significant differences were observed only within the first 5 days of symptom onset, where Ct values were <30 in 38% of saliva samples compared to <10% of nasal samples (Fig 2B).

**Table 3:** Median Ct values of nasal and saliva specimens stratified by days from symptom onset, and results from the Wilcoxon signed-rank tests for the overall analysis cohort.

	n (Pairs)	Nasal VTM		Saliva Direct		Wilcoxon signed-rank test		
Days since symptom onset		Median	IQR	Median	IQR	Median of differences	*Z*	p-value
		Ct		Ct				
**Less than 5 days**	21	34.6	32.3–50.0	32.5	26.6–33.9	8.7	3.02	0.003
**5 to 9 days**	63	40.0	33.75–50.0	35.8	30.9–38.7	4.0	4.58	< 0.001
**10 to 14 days**	35	50.0	39.75–50.0	39.4	32.85–41.5	8.6	2.37	0.003
**15 or more days**	28	50.0	41.88–50.0	38.7	30.82–50.0	6.1	3.14	0.002

#### Nasopharyngeal swabs in VTM versus saliva direct

**Fig 3:**
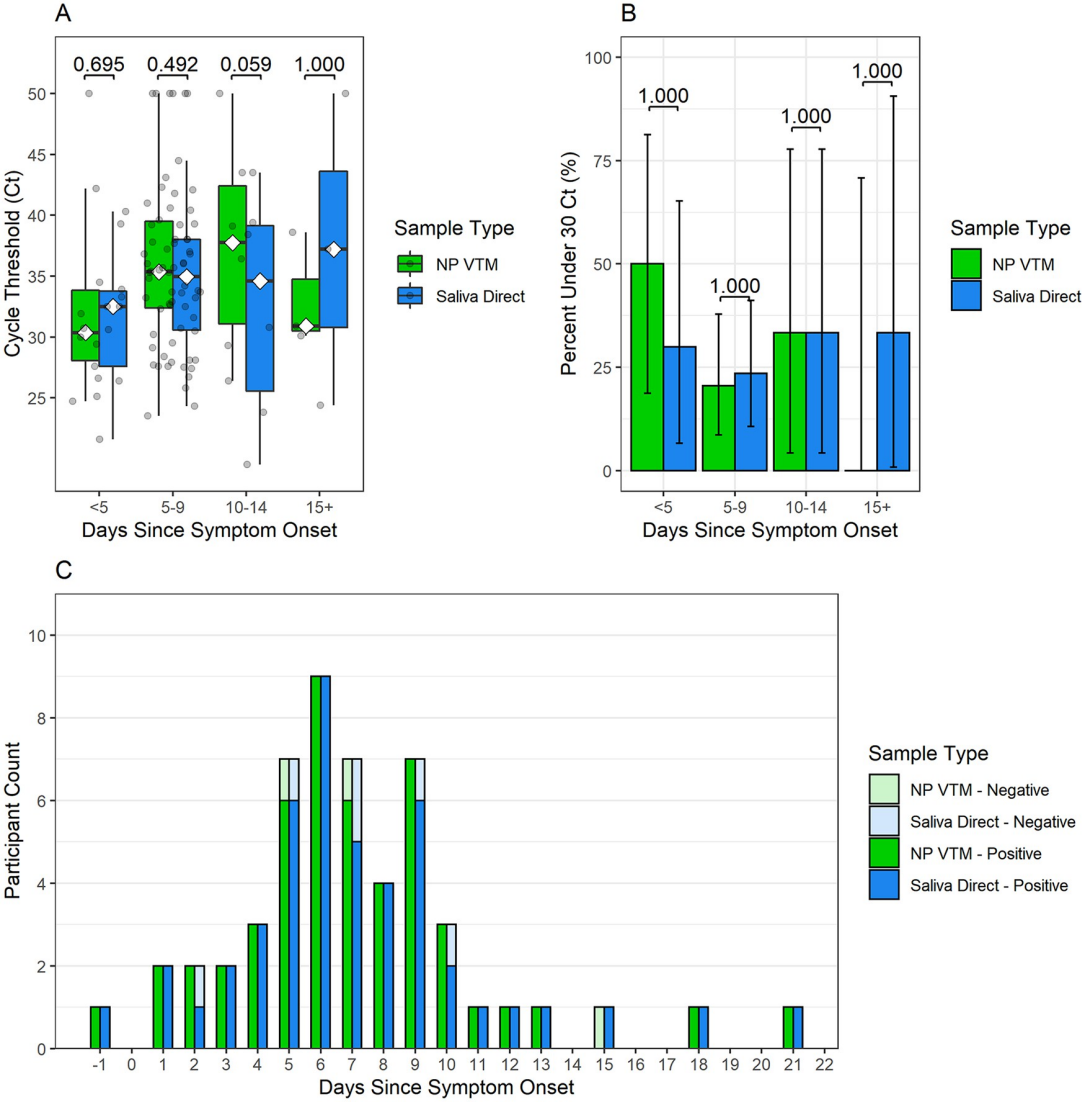
SARS-CoV-2 longevity in overall analysis cohort saliva and nasopharyngeal (NP) specimens. (A) Ct values, (B) percent of Ct values under 30 and (C) number of positive and negative test results for saliva direct and NP VTM specimens by days since symptom onset for all participants in the overall cohort with both saliva and NP at baseline (n = 53).

Median Xpert N2-target Ct values were examined for all patients with both nasopharyngeal (NP) swabs and saliva direct tests at baseline. Positivity rates beyond 5 days from symptom onset were similar between saliva specimens and NP swabs (94.4% [34/36] saliva vs 88.9% [32/36] NP, p-value = 0.683). Positivity rates beyond day 9 of symptoms could not be compared due to a sparsity of paired specimens (n = 6 at 10–14 days; n = 3 beyond 14 days). Overall Ct values were not significantly different between saliva direct (median Ct value = 33.8, 95% CI = 31.6 to 36.8) and NP VTM swabs (median Ct value = 34.8, 95% CI = 31.9 to 36.0) (Fig 3A). Additionally, no differences in median Ct values or in the proportion of tests yielding Ct values under 30 were observed between saliva specimens and NP swabs for any time period (Fig 3B).

#### Controlling for media type and volume differences

**Fig 4:**
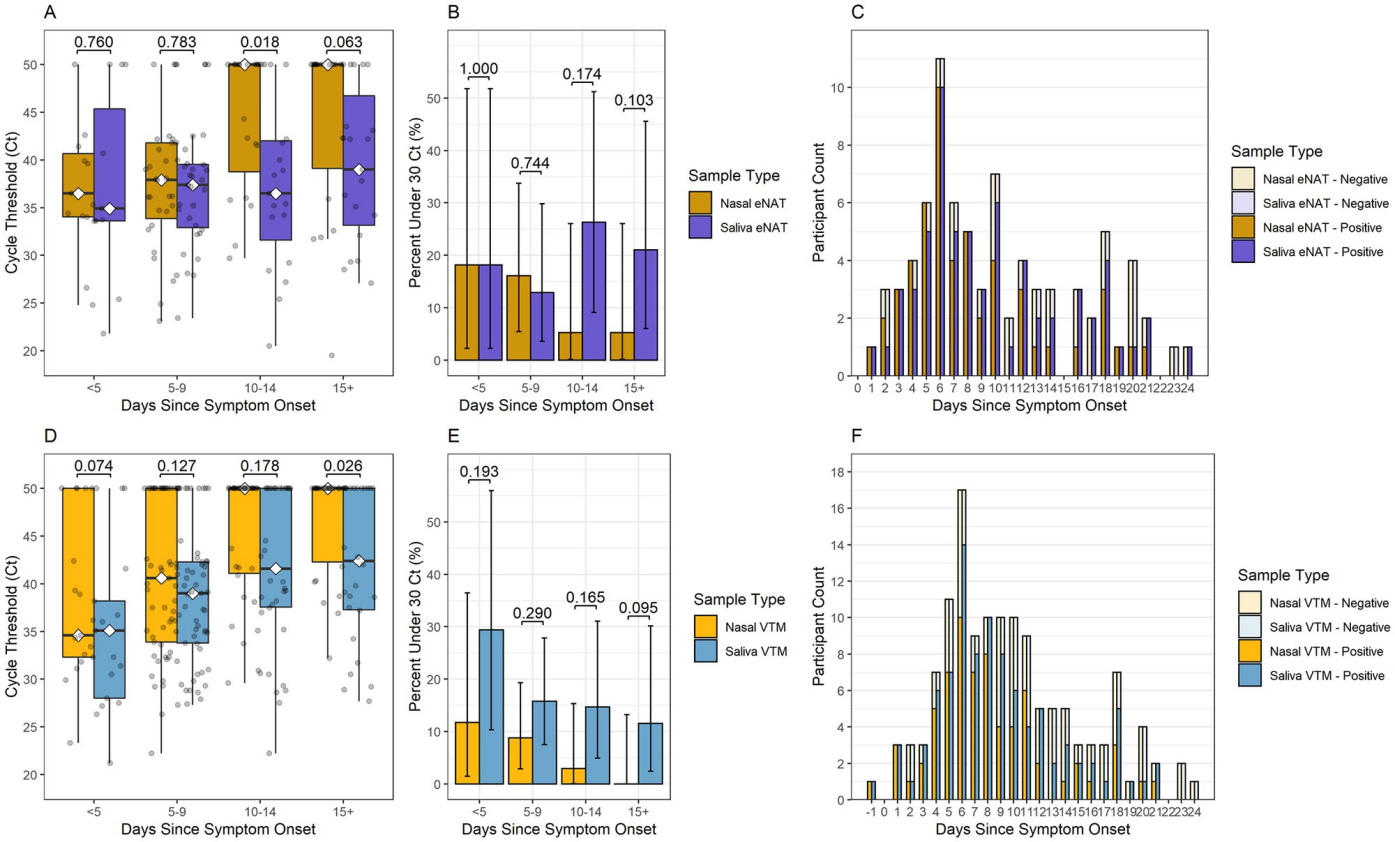
Controlling for media type and volume differences. SARS-CoV-2 longevity in saliva versus nasal specimens in eNAT media, by (A) Ct values, (B) percent of Ct values under 30 and (C) number of positive and negative test results for saliva and nasal swabs over days since symptom onset (n = 80 observations from 45 patients). SARS-CoV-2 longevity in saliva swabbed in VTM media versus nasal swabs in VTM media, by (D) Ct values, (E) percent of Ct values under 30 and (F) number of positive and negative test results for saliva and nasal specimens over time intervals in days since symptom onset (n = 134 observations from 86 patients). Comparisons of medians within each timespan were performed using the Wilcoxon signed-rank test and comparisons of proportions between specimen types within each timespan were performed using the McNemar test for correlated proportions.

We considered a potential confounding effect of specimen volume differences between nasal swabs diluted in VTM and undiluted direct saliva, as well as potential variability across different transport buffers that may increase integrity of RNA preservation. To control for volume differences, we compared the 134 paired nasal and saliva specimens in which the direct saliva sample were swabbed into VTM media from n = 86 of the participants in the overall analysis cohort. To control for differences in transport media, we compared the 80 paired nasal and saliva specimens that were both swabbed into eNAT media from n = 45 of the participants in the overall analysis cohort (Fig 4). No significant difference was observed in N2-Ct values and positivity rates between saliva eNAT and nasal eNAT within 9 days of symptom onset or between saliva VTM and nasal VTM within 14 days of symptom onset. However, saliva continued to have earlier Ct values than their nasal swab counterparts beyond 10 days of symptoms in eNAT (mean Ct of 37.23 vs 44.14, p = 0.018), and beyond 14 days in VTM (mean Ct of 42.23 vs 46.61, p = 0.026). Thus, persistence of viral RNA in saliva at later timepoints was observed even with swabbing both saliva and nasal specimens into the same type and volume of media (Fig 4A).

#### Subgroup analyses

**Fig 5:**
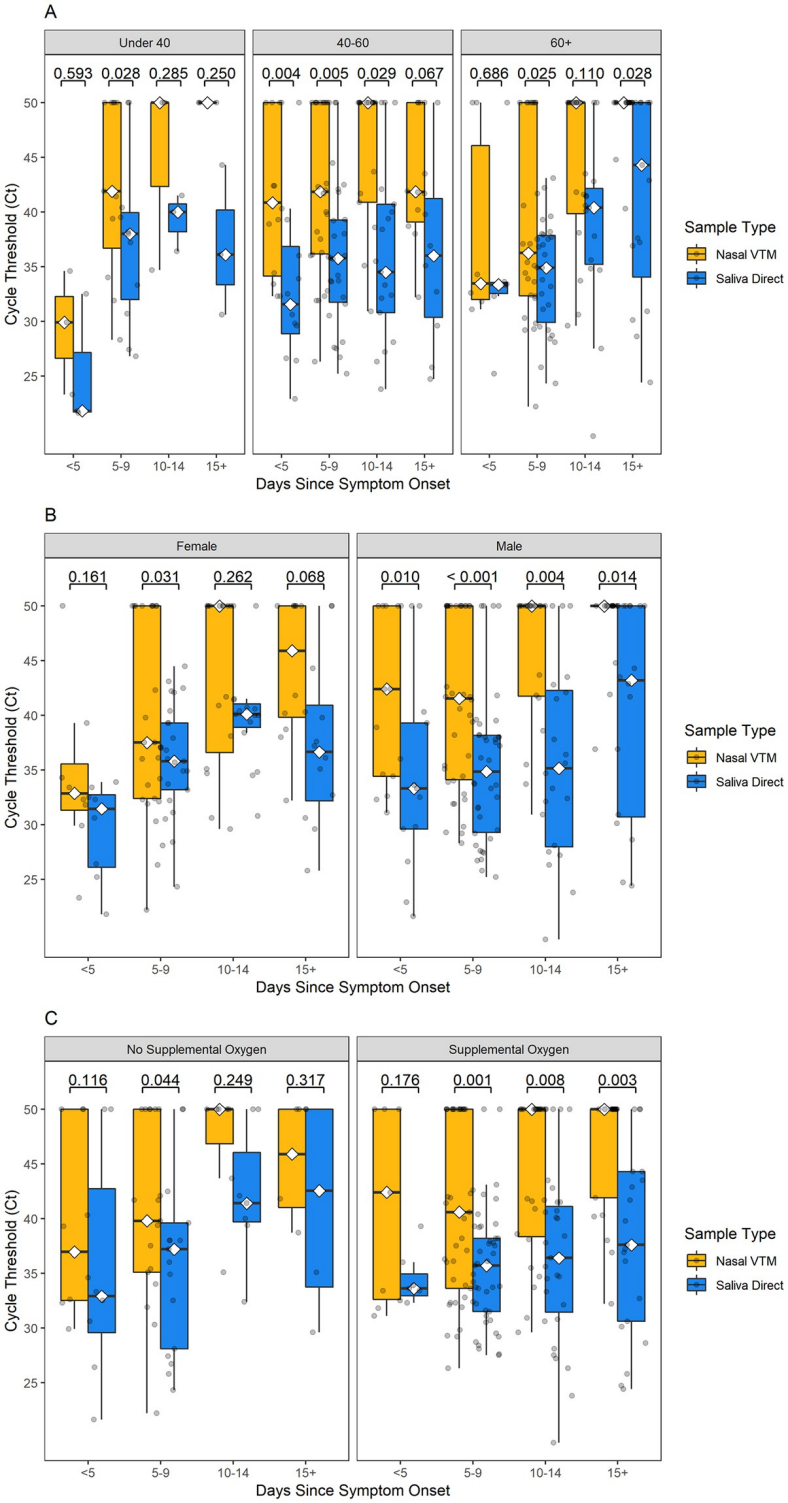
Sub-analysis of SARS-CoV-2 longevity by age, sex, and oxygen requirements. Sub-analysis of Ct values by days since symptom onset for saliva (blue) and nasal (orange) specimens among the overall analysis cohort based on (A) age, (B) sex, and (C) need for oxygen support.

The overall analysis cohort was further characterized by age, sex, and oxygen support status and Ct values were observed across all time intervals. There was a significant to non-significant tendency of lower Ct values (higher viral loads) in saliva compared to nasal swabs across each time interval, for all age (Fig 5A), sex (Fig 5B), and oxygen requirement (Fig 5C) groups.

### Longitudinal analysis

**Fig 6:**
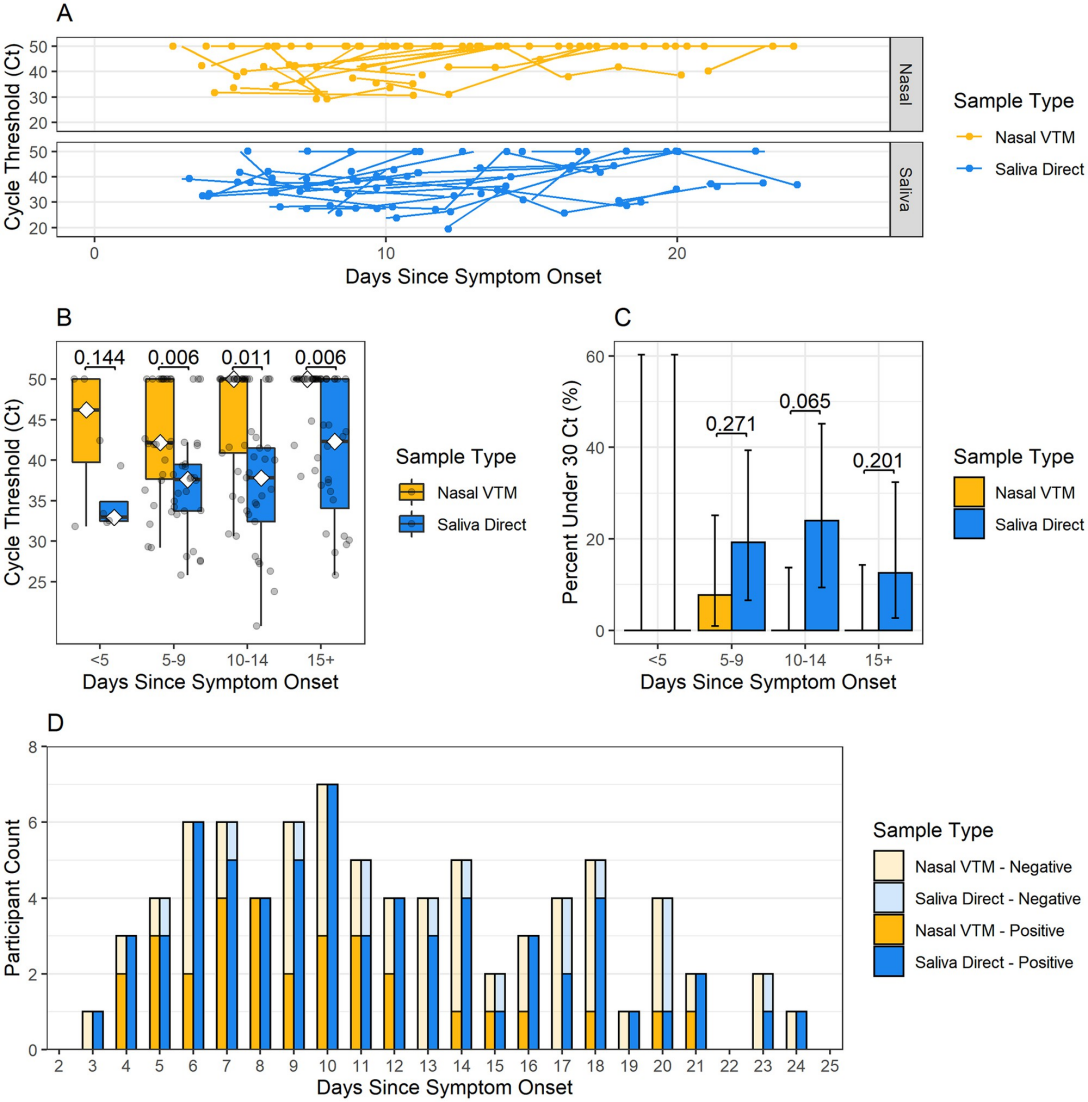
SARS-CoV-2 longevity in longitudinal cohort saliva and nasal specimens. Analysis of paired specimens in the longitudinal analysis cohort (N = 79 paired observations from 28 patients) by (A) Spaghetti plot of longitudinal Ct values for saliva direct and nasal VTM specimens plotted over time in days since symptom onset. Lines indicate specimens from the same patient. (B) Analysis of paired samples in the longitudinal analysis cohort by patient, (B) Ct values, (C) percent of Ct values under 30, and (D) number of positive and negative test results for saliva direct and nasal VTM specimens by days since symptom onset.

**Fig 7:**
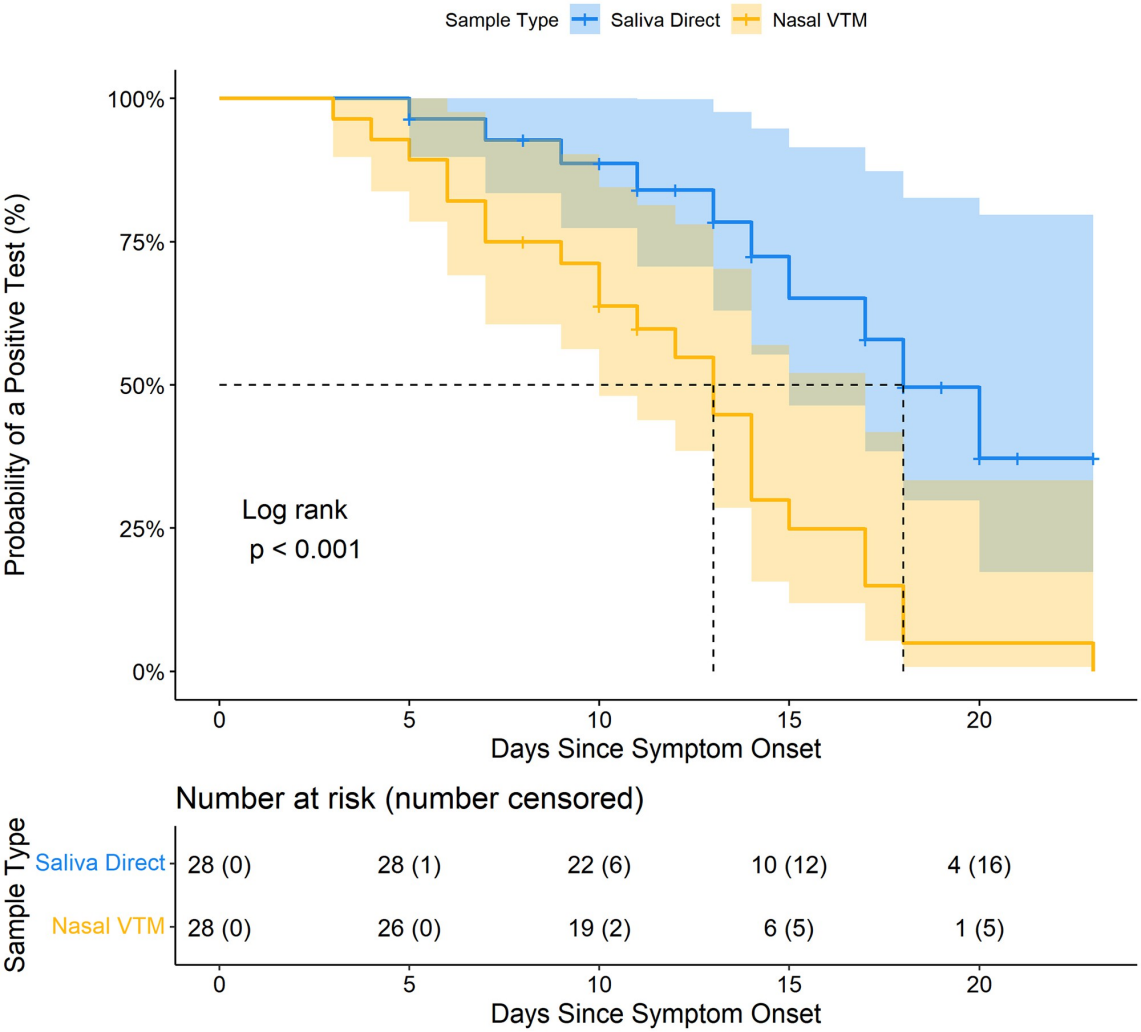
SARS-CoV-2 survival curve in longitudinal cohort saliva and nasal specimens. Kaplan-Meier estimate of probability of a positive test for SARS-CoV-2 in saliva direct and nasal VTM specimens over time in days since symptom onset for the longitudinal cohort. Dashed lines indicate median survival times.

Among the longitudinal analyses cohort (n = 28), substantial fluctuations in viral RNA presence in both saliva and nasal swabs were observed throughout the course of infection (Fig 6A). Across all time periods, earlier median N2-Ct values for saliva were observed compared to nasal swabs, except within 5 days of symptoms represented by only 4 observations (Fig 6B–6D). Saliva also had higher positivity rates than nasal swabs, although this difference could not be statistically estimated with fewer specimens included in the longitudinal analysis (Fig 6C). Kaplan-Meier survival curves for saliva specimens and nasal swabs (Fig 7), and log-rank test indicate that the survival curve for saliva specimens is significantly different from the curve for nasal swabs (p = 0.0007). The median survival time for positive nasal swabs was 13 days, whereas the median survival time for positive saliva specimens was 18 days (Fig 7). The probability of saliva yielding a positive test result remained greater than that of nasal swabs at 10 days (saliva survival probability = 0.89 [95% CI = 0.77–1.00] vs nasal swab survival probability = 0.64 [95% CI = 0.48–0.85]) and 15 days (saliva survival probability = 0.65 [95% CI = 0.46–0.915] vs nasal swab survival probability = 0.25 [95% CI = 0.12–0.52]).

### Viral propagation in saliva beyond 1 week

From the separate UH COVID-19 patient cohort, we detected propagating virus in saliva by TCID50 assays from 3 COVID-19 patients at least 7 days following symptom onset. All 3 patients’ saliva specimens had an RT-PCR Ct value under 30. Characteristics of these patients are shown in S1 Table.

## Discussion

We found that SARS-CoV-2 RNA persists longer and in higher abundance in saliva than in nasal swabs, even beyond 14 days. This has important implications for diagnostic strategies and public health practices. At an individual diagnostic level, these results suggest that testing saliva may be more sensitive than nasal swabs in diagnosing COVID-19 in patients who present during a later disease stage when nasal swabs may become RT-PCR negative. In fact, an important population of hospitalized COVID-19 patients are negative by multiple nasopharyngeal swab RT-PCR tests, leading to missed diagnosis and treatment [1]. Meanwhile several lines of evidence support that collecting and testing multiple specimen types can reduce false-negative COVID-19 diagnosis [14, 24, 25], but saliva is commonly excluded in favor of nasopharyngeal, oropharyngeal, and nasal swab specimens. Our data suggests that testing saliva may reduce false-negative diagnoses in a subset of individuals who present or test later during their course of infection [1], thereby prompting appropriate treatment for these individuals who may have otherwise been missed by swab based testing.

From a public health perspective, the prolonged SARS-CoV-2 RNA detection period combined with observed cases of propagating SARS-CoV-2 in saliva beyond 1 week of symptoms suggests prolonged infectiousness may occur from an oral source. Oral transmission during this prolonged infectiousness period is likely perpetuated by aerosolizing activities such as speaking, coughing, laughing, and singing. These observations provoke considerations for follow up saliva-based testing, and extended mask usage and precautionary measures in individuals who test positive. Whereas follow-up testing is more feasible with rapid antigen tests, further studies are needed to conclude whether the higher proportion of saliva samples with a Ct < 30 beyond the first week of symptoms associate with a higher yield of saliva-based rapid antigen tests [26]. To estimate a probability of infectious potential from oral sources, a larger systematic study correlating viral propagation with Ct values in oral secretions of COVID-19 patients is required, though these studies are labor intensive.

There were several limitations of this study. First, the study was conducted in 2020–2021 prior to emergence of the Omicron variant. However, nasal viral load decay rates in our study are similar to that of Delta and Omicron variants in more recent studies [27], suggesting that nasal viral dynamics later in infection are not widely different between variants. Regardless, these findings support a premise and relevance for understanding differences in oral, nasal, and nasopharyngeal shedding dynamics as new variants emerge to guide testing and public health strategies. Our sample size was small, which limited the power of the study especially for observations beyond 10 days. Similarly, we have few longitudinal data due in part because patients who stayed in the hospital were often more ill and either did not feel well enough to continue the study or could not produce a saliva specimen. Persistence of viral RNA in nasal [25], nasopharyngeal [26], and “respiratory” [8, 27] samples appears directly related to increasing disease severity, though little is known about the relative persistence in saliva in more mild versus more severe cases. Additionally, this study is limited by lack of longitudinal NP swab data as many participants were unwilling to undergo a repeat NP swab collection (beyond what they provided for routine clinical care). However, this in itself reflects the tractability and feasibility of serial saliva testing compared to serial NP swab testing to monitor prolonged shedding.

We found evidence of prolonged presence of SARS-CoV-2 in saliva compared to nasal swabs beyond the first week of symptoms, including propagating virus. These findings support the inclusion of saliva testing later in the disease course to increase the yield of COVID-19 diagnosis, and the consideration of a prolonged oral infectious period in guidance for masking and follow-up saliva-based testing in individuals with known COVID-19.

## Supporting information

S1 Table Detection of propagating SARS-CoV-2 in saliva beyond 1 week. Characteristics of COVID-19 patients from a separate cohort with Ct value < 30 and positive SARS-CoV-2 viral cultures at or beyond 7 days of symptoms.
(DOCX)

S1 Dataset (XLSX)
