# Supplementary material for: Persistence of SARS-CoV-2 in saliva: Implications for late-stage diagnosis and infectious duration
Source: PLoS One. 2023 Mar 16;18(3):e0282708. doi: 10.1371/journal.pone.0282708 (PMC10019618; doi:10.1371/journal.pone.0282708)
Supplement: S1 Table — Characteristics of COVID-19 patients from a separate cohort with Ct value < 30 and positive SARS-CoV-2 viral cultures at or beyond 7 days of symptoms. (DOCX) [file pone.0282708.s001.docx]

|  | Demographics | Symptom duration (days) | Required oxygen support (severe COVID-19)? | Immuno-compromising conditions? | Received any COVID-19 vaccine? | Saliva N2-Ct value | Cytopathic effects in TCID50 |
| --- | --- | --- | --- | --- | --- | --- | --- |
| 1 | 30/Male/Hispanic | 10 | Yes | None known | No | 23.6 | Positive |
| 2 | 53/Female/Black | 28 | Yes | None known | No | 19.7 | Positive |
| 3 | 30/Male/Black | 7 | Yes | None known | No | 20.6 | Positive |

**S1 Table. Detection of propagating SARS-CoV-2 in saliva beyond 1 week.** Characteristics of COVID-19 patients from a separate cohort with Ct value < 30 and positive SARS-CoV-2 viral cultures at or beyond 7 days of symptoms.
